# Association between motoric cognitive risk syndrome and frailty among older Chinese adults

**DOI:** 10.1186/s12877-020-01511-0

**Published:** 2020-03-19

**Authors:** Shanshan Shen, Xingkun Zeng, Liyu Xu, Lingyan Chen, Zixia Liu, Jiaojiao Chu, Yinghong Yang, Xiushao Wu, Xujiao Chen

**Affiliations:** Department of Geriatric, Zhejiang Hospital, No. 12 Lingyin Road, Hangzhou, 310013 Zhejiang Province China

**Keywords:** Cognitive complaints, Slow gait, Frailty, Older adult

## Abstract

**Background:**

Motoric cognitive risk syndrome (MCR) is a newly proposed predementia syndrome incorporating subjective cognitive complaints and slow gait. Previous studies have reported that subjective cognitive complaints and slow gait are associated with frailty in cognitively unimpaired older adults, but little is known about the link between MCR and frailty in older adults. Therefore, the aim of the study was to explore the associations of MCR and its components with frailty in older Chinese adults.

**Methods:**

In an observational cross-sectional study, a total of 429 older adults aged 60 years and older were admitted to the geriatric department. According to MCR criteria, all participants were classified into 4 groups: 1) the MCR group; 2) the subjective cognitive complaints only group; 3) the slow gait only group; and 4) the healthy control group. Physical frailty was assessed by the Clinical Frailty Scale (CFS). Multivariate logistic regression analysis was used to examine the association between MCR and frailty in older adults.

**Results:**

The prevalence rates of subjective cognitive complaints, slow gait and MCR were 15.9, 10.0 and 4.0%, respectively. After adjusting for confounding variables, the logistic regression analysis showed that slow gait (odds ratio [OR]: 3.40, 95% confidence interval [CI]: 1.40–8.23, *P* = 0.007) and MCR (OR: 5.53, 95% CI: 1.46–20.89, *P* = 0.012) were independently associated with frailty, but subjective cognitive complaints were not.

**Conclusions:**

MCR and slow gait were significantly associated with frailty in older Chinese adults. Further studies should prospectively determine the causal relationship between MCR and frailty.

## Background

Accelerated population ageing and increased average life expectancy in China are expected to further increase age-associated pathological conditions, such as cognitive impairment and frailty. Motoric cognitive risk syndrome (MCR) is a predementia syndrome that is characterized by the simultaneous presence of subjective cognitive complaints and slow gait in older individuals without dementia or mobility disability [1]. The reported prevalence of MCR ranged from 2 to 18% [2–7]. Based on the current studies, the lowest MCR prevalence was found in the Australian (2%) and United Kingdom (2%) populations; high prevalence rates were found in Japan (6.4%), China (9.6%), Mexico (14.6%) and India (15%); and the highest prevalence was seen in France (16–18%) studies [2–7]. MCR was found to be associated with adverse clinical outcomes in older individuals, such as dementia, falls, disability and mortality [1, 8–11]. A prospective cohort study based in the Einstein Aging Study showed that older adults with MCR had a greater than 3-fold risk of developing dementia and a greater than 12-fold risk of vascular dementia [1]. A recent study from a Japanese community-dwelling sample of older adults reported that MCR was a risk factor for future dementia (HR = 2.49) and disability (HR = 1.69) [8]. Kumai and colleagues found that the rate of conversion to dementia in the MCR group was 1.38-times higher than that in the non-MCR group, and both slow gait and lower scores on executive tests were reported to be predictive of a higher rate of conversion to dementia [12]. In addition, because screening for MCR does not require complex neuropsychological tests or neuroimaging examinations, it is more conducive to increasing the accessibility of clinical dementia risk assessment and implementing appropriate prevention strategies. Therefore, MCR has increasingly gained clinical attention.

Frailty is a common geriatric syndrome that is partially reversible and characterized by age-related decline in physiologic reserves and function of multiple systems, resulting in increasing vulnerability triggered by minor stressor events and further leading to negative health outcomes, including falls, disability, hospitalization and mortality [13–15]. A growing body of evidence has shown that cognitive impairment and frailty are closely related in populations with advanced age in that they may share similar etiologies. For example, frail status may increase the risk of cognitive decline, incident mild cognitive impairment and dementia [16–20]. Alternatively, cognitive impairment also increases the risk of frailty [21, 22]. Frailty and cognitive impairment often coexist [23, 24], so the construct of cognitive frailty was proposed, which is also described as a risk factor for dementia [25, 26].

Compared with the operational definition of cognitive frailty incorporating physical frailty and cognitive impairment without an overt dementia diagnosis, MCR focuses more on subjective cognitive complaints but not mild cognitive impairment (MCI) [27]. Subjective cognitive complaints with normal objective cognitive test results indicate the presence of subjective cognitive decline [28]. Subjective cognitive decline has been reported to be predictive of incident MCI [29], as well as dementia [30]. Thus, MCR might represent an earlier stage of preclinical dementia. Additionally, although subjective cognitive decline and slow gait are reported to be associated with frailty in cognitively unimpaired older adults [24, 25, 31], little is known about the link between MCR and frailty in older adults. Based on these studies, we hypothesized that older adults with MCR may exhibit a higher risk of frailty than older adults with subjective cognitive complaints or slow gait. Therefore, we conducted this study to explore the associations of MCR and its components with frailty in older Chinese adults.

## Methods

### Participants

An observational cross-sectional study was carried out with a total of 935 potential participants from the geriatric department of Zhejiang Hospital in China, and the baseline assessments were performed from October 2014 to September 2018. The inclusion criteria in this study were an age of 60 years or older and the ability to understand and communicate in Chinese. Participants with a history of Parkinson’s disease, Parkinson’s syndrome, dementia, MCI indicated by a score lower than 24 on the Mini-Mental State Examination (MMSE) or mobility disability [1] who were unable to ambulate with or without walking aids were excluded. Participants who had incomplete data used to diagnose MCR were also excluded. Data were collected by a trained geriatric physician and nurse via a computer-aided hospital-based comprehensive geriatric assessment.

Approval for this study was granted by the medical ethics committees of Zhejiang Hospital (2013–25), and written informed consent was obtained from each participant. This study has not been registered, and the manuscript was written according to the STrengthening the Reporting of OBservational studies in Epidemiology (STROBE) statement.

### MCR diagnosis

Based on the MCI operational definition [32, 33], Verghese and colleagues proposed the MCR concept [1]. MCR was diagnosed as both the presence of subjective cognitive complaints and slow gait in those without dementia or mobility disability [1]. Subjective cognitive complaints were determined by face-to-face interviews based on responses to one item on a 15-item geriatric depression scale (GDS-15) [34]. The standardized question “Do you feel you have more problems with memory than most?” was asked by a well-trained nurse. A positive response “yes” on this question indicated a subjective cognitive complaint. Gait speed (m/s) was calculated by the four-metre usual gait speed test. The participants completed the test twice while starting from an inactive standing position (walking aids or cane allowed), and the shortest time was recorded. Slow gait was defined as gait speed one standard deviation below the age- and sex-specific means [1]. The cut-off values of slow gait in this study were: males 60–74 years ≤0.91 m/s, males ≥75 years ≤0.69 m/s, females 60–74 years ≤0.80 m/s, females ≥75 years ≤0.66 m/s.

According to the MCR criteria, all participants were classified to 4 groups: 1) the MCR group; 2) the subjective cognitive complaints only group; 3) the slow gait only group; and 4) the healthy control group.

### Frailty assessment

Based on the Canadian study on Health and Aging, frailty was assessed by the Clinical Frailty Scale (CFS), which was scored from 1 (very fit) to 7 (severely frail) [35, 36]. The evaluator recorded the level of frailty using their clinical judgement based on available clinical information. In this study, a CFS score greater than four indicated frailty [37].

The CFS was assessed by a well-trained assessor who was qualified in geriatric comprehensive assessment. There are three qualified members in our team, and they had evaluated a total of 1122 cases. The CFS assessor was blinded to the MCR assessor results.

#### Other covariates

Demographic data including age, sex, educational level, marital status, cigarette smoking and alcohol drinking use were obtained. Smoking status was categorized into non-smokers, former smokers and current smokers on the basis of self-reported amount and length of cigarette smoking. Current smokers were those who smoked regularly at least once a day or more for more than half a year, and former smokers were those who used to smoke but stopped smoking at least half a year prior to the study [38]. Alcohol drinking status was classified into non-drinkers, former drinkers and current drinkers based on the self-reported amount and duration of alcoholic beverages. Current drinkers were those who drank at least once a week for more than 6 months, and former drinkers were defined those who stopped drinking at least 6 months prior to the start of the study [38]. Body mass index (BMI) was calculated with height and weight. Physician-diagnosed chronic medical diseases were recorded according to the International Classification of Diseases, Tenth Revision (ICD-10) codes. Diabetes mellitus, cerebrovascular diseases, hypertension and coronary artery disease were included in this study. Comorbidities were defined as the coexistence of five kinds of chronic diseases or more. Participants who took five or more oral prescription medications were considered to the polypharmacy criteria [39]. A history of falls in the past year was also recorded. A fall was defined as a sudden, involuntary, unintentional change of position resulting in rest on the ground or another lower plane [40]. Dominant hand grip strength was measured three times using a hand dynamometer. The maximum of the three measurements was recorded for the final analysis. Depression symptoms were evaluated using the GDS-15 [34] and cognitive function was assessed using the MMSE [41].

### Statistical analysis

Normal distributed continuous variables are presented as the means± standard deviations (SDs), and categorical variables are expressed as numbers (percentages). The unpaired t-test (for normally distributed continuous data) and the chi-square test (for categorical data) were used to identify the significant differences between the male and female groups. One-way ANOVA (for normally distributed continuous data) and the chi-square test (for categorical data) were used to identify the significant differences among the control, subjective cognitive complaints only, slow gait only and MCR groups. Furthermore, the association of MCR with frailty was analysed using multivariate logistic regression models and expressed in odds ratios (ORs) and 95% confidence intervals (CIs). Multivariate logistic regression was conducted with 3 models. Model 1 was not adjusted covariates; Model 2 was adjusted for age, sex and education; and Model 3 was adjusted for marital status, BMI, comorbidities, polypharmacy, fall history, grip strength, depressive symptoms and MMSE scores, in addition to the variables adjusted in Model 2. The data were analysed by using SPSS 18.0 software (SPSS, Chicago, IL, USA). All significance tests were two-tailed, and statistical significance was indicated by *P <* 0.05.

## Results

### Enrolment, prevalence rates and characteristics of the total sample

**Fig. 1:**
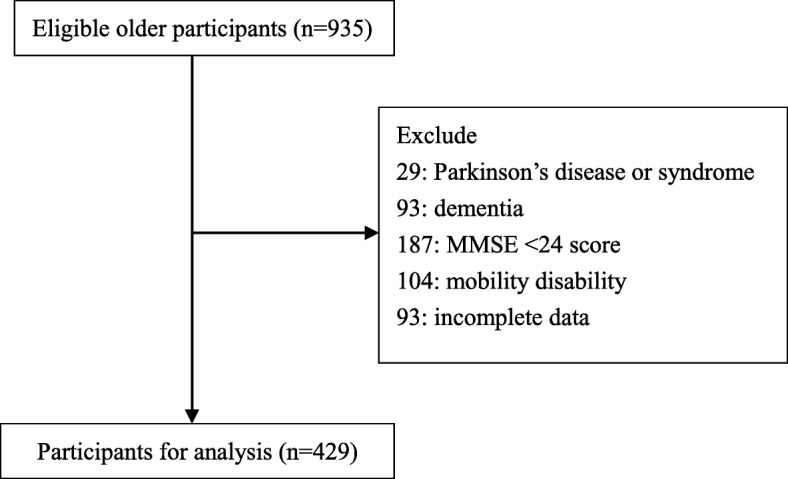
Flow chart of participant selection

Figure 1 shows the flow chart of participant selection. A total of 935 potential participants were screened, and after exclusions, 429 patients were finally eligible.

**Fig. 2:**
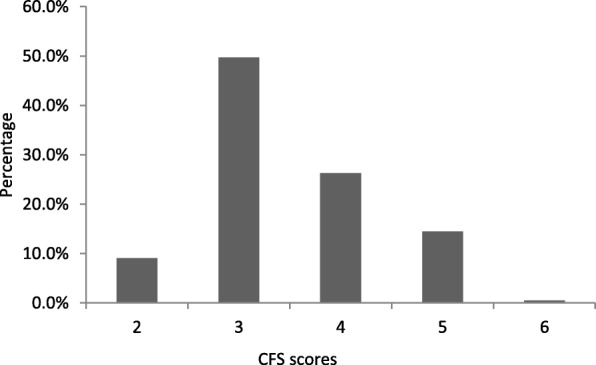
Distribution of CFS scores in the total sample

The characteristics of the 429 participants are presented in Table 1. The mean age of the participants was 76.7 (±7.7) years, 59.9% were male, 66.0% had an educational level of high school or above and 79.3% were married. The mean BMI was 23.7 (±3.2) kg/m^2^. More than half (60.4%) of the participants reported 5 or more comorbid conditions, and hypertension (75.8%) was the most common chronic disease. Nearly half of the participants (46.2%) met the criteria for polypharmacy. Approximately 15% of participants were classified as physically frail. Compared with males, females were older, had a lower educational level, were more often married, had a higher percentage of smoking and drinking history, had lower grip strength and were more likely to have depressive symptoms (all *P* for trend < 0.05), as detailed in Table 1. The distribution of CFS scores is shown in Fig. 2.

**Table 1:** Characteristics and comparing in male and female

	Total (*n* = 429)	Males (*n* = 257)	Females (*n* = 172)	*P-*value
Age, years (mean ± SD)	76.7 ± 7.7	77.4 ± 8.0	75.7 ± 7.2	0.027
High school and above, *n* (%)	283 (66.0)	179 (69.6)	104 (60.5)	0.049
Married, *n* (%)	340 (79.3)	221 (86.0)	119 (69.2)	< 0.001
Current or former smoker, *n* (%)	100 (23.3)	100 (38.9)	0 (0)	< 0.001
Current or former drinker, *n* (%)	99 (23.1)	93 (36.2)	6 (3.5)	< 0.001
BMI (mean ± SD, kg/m^2^)	23.7 ± 3.2	23.8 ± 3.0	23.5 ± 3.5	0.312
History of diabetes, *n* (%)	101 (23.5)	64 (24.9)	37 (21.5)	0.417
History of cerebrovascular diseases, *n* (%)	129 (30.1)	78 (30.4)	51 (29.7)	0.877
History of hypertension, *n* (%)	325 (75.8)	199 (77.4)	126 (73.3)	0.323
History of coronary artery disease, *n* (%)	141 (32.9)	85 (33.1)	56 (32.6)	0.911
Comorbidities (≥ 5 diseases), *n* (%)	259 (60.4)	157 (61.1)	102 (59.3)	0.711
Polypharmacy (≥ 5 drugs), *n* (%)	198 (46.2)	118 (45.9)	80 (46.5)	0.903
Fall history in the past year, *n* (%)	69 (16.1)	41 (16.0)	28 (16.3)	0.928
Grip strength, (mean ± SD, kg)	30.6 ± 9.4	35.5 ± 8.1	23.3 ± 5.5	< 0.001
Four-meter usual gait speed (mean ± SD, m/s)	1.0 ± 0.3	1.0 ± 0.3	1.0 ± 0.3	0.622
Depressive symptoms from GDS-15 (mean ± SD, scores)	2.2 ± 2.4	1.8 ± 2.1	2.9 ± 2.8	< 0.001
MMSE (mean ± SD, scores)	27.4 ± 1.9	27.3 ± 1.9	27.5 ± 1.8	0.145
Frailty for CFS ≥ 5, *n* (%)	64 (14.9)	38 (14.8)	26 (15.1)	0.925

**Abbreviations:***BMI* body mass index, *GDS-15* 15-item Geriatric Depression Scale, *MMSE* Mini-Mental State Examination, *CFS* Clinical Frailty Scale. The *P*-value is for comparing sex differences

Of the total participants, the prevalence rates of subjective cognitive complaints only, slow gait only and MCR were 15.9, 10.0 and 4.0%, respectively.

### Comparing variables between groups

As shown in Table 2, significant differences were observed among the four groups (i.e., the control, subjective cognitive complaints only, slow gait only and MCR groups) with regard to BMI, polypharmacy, grip strength, four-metre gait speed, GDS-15 scores, MMSE scores and physical frailty (all *P* for trend < 0.05). There were no significant differences in age, sex, educational level, marital status, smoking history, alcohol drinking history, comorbid diseases (such as diabetes mellitus, cerebrovascular diseases, hypertension and coronary artery disease) or fall history in the past year among the groups (all *P* for trend > 0.05).

**Table 2:** Characteristics and comparing among the four groups

	Healthy control (*n* = 301)	Subjective cognitive complaints only (*n* = 68)	Slow gait only (*n* = 43)	MCR (n = 17)	*P-*value
Age, years (mean ± SD)	76.6 ± 7.6	75.7 ± 7.8	79.1 ± 7.7	76.7 ± 8.7	0.156
Male, *n* (%)	189 (62.8)	34 (50.0)	27 (62.8)	7 (41.2)	0.092
High school and above, *n* (%)	207 (68.8)	40 (58.8)	28 (65.1)	8 (47.1)	0.150
Married, *n* (%)	239 (79.4)	58 (85.3)	29 (67.4)	14 (82.4)	0.154
Current or former smoker, *n* (%)	73 (24.3)	15 (22.1)	9 (20.9)	3 (17.6)	0.885
Current or former drinker, *n* (%)	74 (24.6)	13 (19.1)	9 (20.9)	3 (17.6)	0.710
BMI (mean ± SD, kg/m^2^)	23.7 ± 3.0	22.8 ± 3.1	24.5 ± 3.8	24.4 ± 4.5	0.032
History of diabetes, *n* (%)	76 (25.2)	8 (11.8)	13 (30.2)	4 (23.5)	0.079
History of cerebrovascular diseases, *n* (%)	86 (28.6)	19 (27.9)	15 (34.9)	9 (52.9)	0.160
History of hypertension, *n* (%)	223 (74.1)	52 (76.5)	36 (83.7)	14 (82.4)	0.500
History of coronary artery disease, *n* (%)	93 (30.9)	24 (35.3)	15 (34.9)	9 (52.9)	0.273
Comorbidities (≥ 5 diseases), *n* (%)	176 (58.5)	42 (61.8)	29 (67.4)	12 (70.6)	0.542
Polypharmacy (≥ 5 drugs), *n* (%)	132 (43.9)	27 (39.7)	27 (62.8)	12 (70.6)	0.014
Fall history in the past year, *n* (%)	50 (16.6)	10 (14.7)	8 (18.6)	1 (5.9)	0.643
Grip strength, (mean ± SD, kg)	31.8 ± 9.2	29.7 ± 8.8	26.8 ± 9.7	23.0 ± 6.8	< 0.001
Four-meter usual gait speed (mean ± SD, m/s)	1.1 ± 0.2	1.1 ± 0.2	0.6 ± 0.1	0.6 ± 0.2	< 0.001
Depressive symptoms from GDS-15 (mean ± SD, scores)	1.6 ± 2.0	4.0 ± 2.7	2.7 ± 2.4	5.1 ± 2.9	< 0.001
MMSE (mean ± SD, scores)	27.6 ± 1.8	27.2 ± 1.8	26.9 ± 2.0	25.7 ± 1.7	< 0.001
Frailty for CFS ≥ 5, *n* (%)	33 (11.0)	7 (10.3)	16 (37.2)	8 (47.1)	< 0.001

**Abbreviations:***BMI* body mass index, *GDS-15* 15-item Geriatric Depression Scale, *MMSE* Mini-Mental State Examination, *CFS* Clinical Frailty Scale

### The association between MCR and frailty

Table 3 shows the results of the multivariate logistic regression analysis on the association between MCR and frailty. Crude Model 1 showed that slow gait (OR: 4.81, 95% CI: 2.35–9.85) and MCR (OR: 7.22, 95% CI: 2.61–19.99) were associated with frailty. Model 2, after adjusting for age, sex and education, showed that slow gait (OR: 4.36, 95% CI: 1.94–9.80) and MCR (OR: 9.98, 95% CI: 2.88–34.59) remained associated with frailty. In the fully adjusted Model 3, the results remained unchanged, and slow gait (OR: 3.40, 95% CI: 1.40–8.23) and MCR (OR: 5.53, 95% CI: 1.46–20.89) were associated with an increased risk of frailty. However, no significant association was found between subjective cognitive complaints and frailty.

**Table 3:** Multivariate analysis of the association between MCR and physical frailty

	Model 1		Model 2		Model 3	
	OR(95%CI)	*P-*value	OR(95%CI)	*P-*value	OR(95%CI)	*P-*value
Healthy control	1.00	–	1.00	–	1.00	–
Subjective cognitive complaints only	0.93 (0.39–2.21)	0.873	1.03 (0.41–2.56)	0.952	0.76 (0.26–2.26)	0.619
Slow gait only	4.81 (2.35–9.85)	< 0.001	4.36 (1.94–9.80)	< 0.001	3.40 (1.40–8.23)	0.007
MCR	7.22 (2.61–19.99)	< 0.001	9.98 (2.88–34.59)	< 0.001	5.53 (1.46–20.89)	0.012

**Abbreviations:***OR* odd ratio, *CI* confidence interval, *BMI* body mass index

**Notes:** Model 1: crude model; Model 2: adjusted for age, sex and education; Model 3: adjusted for Model 2 plus BMI, comorbidities, polypharmacy, grip strength, depressive symptoms and MMSE scores

## Discussion

In this study, MCR and slow gait were both significantly associated with frailty in older adults in the geriatric department, but subjective cognitive complaints were not associated with frailty. These associations remained unchanged after adjusting for potential confounding variables. These results revealed that the association between MCR and frailty was commonly observed along with slow gait, and the combination of subjective cognitive complaints and slow gait may have greater predictive value than the single component to explain their common association with frailty.

The primary finding of this study is that MCR was associated with frailty in elderly population. This finding is in accordance with a cross-sectional study from France [42], which showed that physical frailty and, in particular, slow gait speed were associated with cognitive impairment, indirectly reflecting that slow gait and cognitive impairment often coexist with MCR and suggesting that MCR and frailty interact with each other in the context of ageing. Subjective cognitive complaints and slow gait are common conditions in the ageing process and have been considered early clinical indicators of cognitive impairment and dementia during the preclinical stages. Cognitive impairment is a longitudinal process that begins with minor alterations in certain domains and slowly progresses to multiple changes in many domains [43]. The accumulation of cerebral amyloid deposition and neurofibrillary tangles plays a critical role in the progression of Alzheimer’s disease neuropathology [44, 45]. In addition, alterations in brain regions, including the motor cortices, striatum and substantia nigra are often involved [44, 45]. Studies have shown that MCR involves a wider range of brain regions and that its involvement is not limited to the hippocampus, which is a key brain region for memory and spatial navigation processes [46–48]. MCR has been characterized by smaller volumes of total grey matter, total cortical grey matter, the premotor cortex, and the prefrontal cortex, especially the dorsolateral segment; however, no significant differences were found in terms of the volumes of hippocampal and white matter [49, 50], which is in concordance with these results as these two components control cognitive complaints and slow gait. MCR-related brain region reduction contributes more to the prediction of cortical neurodegenerative dementia than that of subcortical dementia, such as vascular dementia [49]. A study by Wang and colleagues found that frontal lacunar infarcts were associated with slow gait, poor memory function and MCR, but not with cognitive complaints [51], revealing that this brain area might result in MCR by disrupting frontally based neural networks facilitating memory and gait functions [52, 53]. On the other hand, the accumulation of common brain pathologies and related brain area alterations may also contribute to progressive frailty [54, 55]. Cerebral amyloid-beta deposition and neurofibrillary tangles were associated with symptoms of frailty, including slower gait speed and lower body mass index in dementia-free elderly individuals [56, 57]. Thus, the association between MCR and frailty could be explained by the abovementioned mechanisms. Slow gait speed is an indicator of physical frailty in the elderly population, which can explain the association between slow gait and frailty in our samples. However, the lack of longitudinal prospective studies of the causal associations between MCR and frailty prevents conclusions from being drawn.

The association between subjective cognitive complaints and frailty was not observed in this study sample. This finding was inconsistent with the results of previous studies, such as the Hellenic Longitudinal Investigation of Aging and Diet study [31] and the Healthy Aging Longitudinal Study in Taiwan [24], revealing that subjective cognitive decline was associated with an increased likelihood of frailty in cognitively unimpaired elderly individuals. Self-reported measures of subjective cognitive decline complaints were adopted, but discrepant results were obtained due to the differences in frailty assessment criteria to determine frailty status. In addition, the characteristics of the enrolled study sample were inconsistent. The potential neurobiological explanations for the association between subjective cognitive decline and frailty include that both factors increase the risk of future cognitive decline [16, 17, 29, 30] and Alzheimer’s disease pathology [58–61].

An important advantage of this study is that all the enrolled older participants underwent a comprehensive geriatric assessment focusing on the functional status of elderly individuals, such as activities of daily living, cognition and frailty. Indeed, in our geriatric department, we have a trained team for comprehensive geriatric assessment, and all the assessments are designed to be conducted with a professional software system. Thus, the validity and consistency of the evaluations are guaranteed. Another advantage is that MCR and CFS-defined frailty screening in this study are relatively simple and easy to implement in other clinical settings, such as community health service centres and other primary medical centres. CFS was reported to reflect the dynamic and reversible changes in frailty and was strongly associated with the frailty index. Thus, it can similarly predict adverse outcomes for elderly patients [35, 62]. In contrast, this study also has some limitations. First, although this study is the first to report the association between MCR and frailty in an older Chinese population, the cross-sectional nature of study precluded us from elucidating the causal relationships. Second, due to the use of a convenience sampling method for the recruitment of inpatients in a monocentric study and the relatively small sample size of MCR cases, there are selection and a representativeness biases in this study. Third, although these study participants were in the normal MMSE range, some participants with mild hidden dementia might be undiagnosed. Finally, the standard definition of MCR is also a limitation of this study. The definition of slow gait is already standardized, but subjective cognitive complaints have not been standardized. Subjective cognitive complaints were defined by one item of the GDS-15 in this study according to the standard method in most studies. Furthermore, the accuracy of self-reported cognitive complaints may be affected by the disease, emotional and environmental factors. Thus, the generalization of the study results could not be assumed because the results are less sensitive for non-demented older adults.

## Conclusions

Our results support the idea that MCR and slow gait were associated with frailty in older adults without dementia or mobility disability. Further study is needed to better understand the pathophysiological, behavioural and environmental variables to prospectively determine the causal relationship between MCR and frailty in a multicenter study with a large sample. More research is also necessary to develop new interventions and preventative strategies for improving quality of life and reducing or reversing cognitive impairments and frailty in older adults.

## Data Availability

The datasets used and/or analysed during the current study are available from the corresponding author on reasonable request.

## Abbreviations

MCR: Motoric cognitive risk
CFS: Clinical Frailty Scale
MCI: Mild cognitive impairment
BMI: Body mass index
MMSE: Mini-Mental State Examination
GDS-15: 15-item Geriatric Depression Scale
SD: Standard deviation
OR: Odds ratio
CI: Confidence interval
